# The GSA Family in 2025: A Broadened Sharing Platform for Multi-omics and Multimodal Data

**DOI:** 10.1093/gpbjnl/qzaf072

**Published:** 2025-08-26

**Authors:** Sisi Zhang, Xu Chen, Enhui Jin, Anke Wang, Tingting Chen, Xiaolong Zhang, Junwei Zhu, Lili Dong, Yanling Sun, Caixia Yu, Yubo Zhou, Zhuojing Fan, Huanxin Chen, Shuang Zhai, Yubin Sun, Qiancheng Chen, Jingfa Xiao, Shuhui Song, Zhang Zhang, Yiming Bao, Yanqing Wang, Wenming Zhao

**Affiliations:** National Genomics Data Center, China National Center for Bioinformation, Beijing 100101, China; Beijing Institute of Genomics, Chinese Academy of Sciences, Beijing 100101, China; National Genomics Data Center, China National Center for Bioinformation, Beijing 100101, China; Beijing Institute of Genomics, Chinese Academy of Sciences, Beijing 100101, China; National Genomics Data Center, China National Center for Bioinformation, Beijing 100101, China; Beijing Institute of Genomics, Chinese Academy of Sciences, Beijing 100101, China; University of Chinese Academy of Sciences, Beijing 100049, China; National Genomics Data Center, China National Center for Bioinformation, Beijing 100101, China; Beijing Institute of Genomics, Chinese Academy of Sciences, Beijing 100101, China; National Genomics Data Center, China National Center for Bioinformation, Beijing 100101, China; Beijing Institute of Genomics, Chinese Academy of Sciences, Beijing 100101, China; National Genomics Data Center, China National Center for Bioinformation, Beijing 100101, China; Beijing Institute of Genomics, Chinese Academy of Sciences, Beijing 100101, China; National Genomics Data Center, China National Center for Bioinformation, Beijing 100101, China; Beijing Institute of Genomics, Chinese Academy of Sciences, Beijing 100101, China; National Genomics Data Center, China National Center for Bioinformation, Beijing 100101, China; Beijing Institute of Genomics, Chinese Academy of Sciences, Beijing 100101, China; National Genomics Data Center, China National Center for Bioinformation, Beijing 100101, China; Beijing Institute of Genomics, Chinese Academy of Sciences, Beijing 100101, China; National Genomics Data Center, China National Center for Bioinformation, Beijing 100101, China; Beijing Institute of Genomics, Chinese Academy of Sciences, Beijing 100101, China; National Genomics Data Center, China National Center for Bioinformation, Beijing 100101, China; Beijing Institute of Genomics, Chinese Academy of Sciences, Beijing 100101, China; National Genomics Data Center, China National Center for Bioinformation, Beijing 100101, China; Beijing Institute of Genomics, Chinese Academy of Sciences, Beijing 100101, China; National Genomics Data Center, China National Center for Bioinformation, Beijing 100101, China; Beijing Institute of Genomics, Chinese Academy of Sciences, Beijing 100101, China; National Genomics Data Center, China National Center for Bioinformation, Beijing 100101, China; Beijing Institute of Genomics, Chinese Academy of Sciences, Beijing 100101, China; National Genomics Data Center, China National Center for Bioinformation, Beijing 100101, China; Beijing Institute of Genomics, Chinese Academy of Sciences, Beijing 100101, China; National Genomics Data Center, China National Center for Bioinformation, Beijing 100101, China; Beijing Institute of Genomics, Chinese Academy of Sciences, Beijing 100101, China; National Genomics Data Center, China National Center for Bioinformation, Beijing 100101, China; Beijing Institute of Genomics, Chinese Academy of Sciences, Beijing 100101, China; University of Chinese Academy of Sciences, Beijing 100049, China; National Genomics Data Center, China National Center for Bioinformation, Beijing 100101, China; Beijing Institute of Genomics, Chinese Academy of Sciences, Beijing 100101, China; University of Chinese Academy of Sciences, Beijing 100049, China; National Genomics Data Center, China National Center for Bioinformation, Beijing 100101, China; Beijing Institute of Genomics, Chinese Academy of Sciences, Beijing 100101, China; University of Chinese Academy of Sciences, Beijing 100049, China; National Genomics Data Center, China National Center for Bioinformation, Beijing 100101, China; Beijing Institute of Genomics, Chinese Academy of Sciences, Beijing 100101, China; University of Chinese Academy of Sciences, Beijing 100049, China; National Genomics Data Center, China National Center for Bioinformation, Beijing 100101, China; Beijing Institute of Genomics, Chinese Academy of Sciences, Beijing 100101, China; National Genomics Data Center, China National Center for Bioinformation, Beijing 100101, China; Beijing Institute of Genomics, Chinese Academy of Sciences, Beijing 100101, China; University of Chinese Academy of Sciences, Beijing 100049, China

**Keywords:** High-throughput sequencing data, Multi-omics data, Data archiving, Data sharing, Genome Sequence Archive

## Abstract

The Genome Sequence Archive family (GSA family) provides a comprehensive suite of database resources for archiving, retrieving, and sharing multi-omics data for the global academic and industrial communities. It currently comprises four distinct database members: the Genome Sequence Archive (GSA, https://ngdc.cncb.ac.cn/gsa), the Genome Sequence Archive for Human (GSA-Human, https://ngdc.cncb.ac.cn/gsa-human), the Open Archive for Miscellaneous Data (OMIX, https://ngdc.cncb.ac.cn/omix), and the Open Biomedical Imaging Archive (OBIA, https://ngdc.cncb.ac.cn/obia). Compared to its 2021 version, the GSA family has expanded significantly by introducing a new repository, the OBIA, and by comprehensively upgrading the existing databases. Notable enhancements to the existing members include broadening the range of accepted data types, strengthening quality control systems, improving the data retrieval system, and refining data-sharing management mechanisms.

## Graphical abstract

**Figure:**
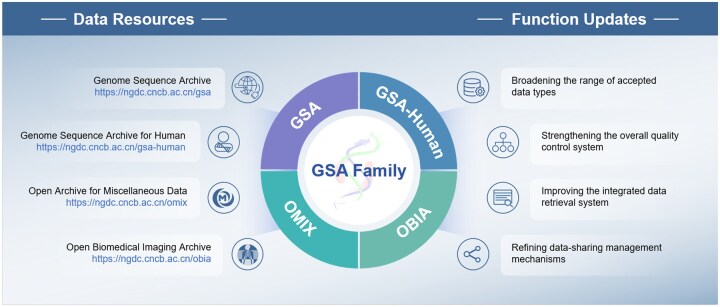

## Introduction

Next-generation sequencing (NGS) technologies have revolutionized genomics, leading to an exponential increase in the amount of available raw sequence data and expanding researchers’ understanding of genome structure, function, and complexity [1,2]. The advancements in high-throughput omics technologies have empowered researchers to adopt multi-omics strategies that integrate and analyze data from different layers, including genomics, transcriptomics, epigenomics, proteomics, and metabolomics, thereby deepening our understanding of complex biological phenomena [3–5]. In recent years, advances in medical imaging technologies, such as magnetic resonance imaging (MRI), computed tomography (CT), and positron emission tomography (PET), along with improvements in image analysis, have enabled the extraction of detailed imaging features. These features can now be integrated with multi-omics data, opening new avenues for medical research and personalized healthcare [6–8]. These factors collectively have driven the transition from single-omics to multi-omics research and have advanced data management toward standardization, normalization, and platform-based sharing. The management and sharing of multi-omics and multimodal data present unique complexities, primarily in the following aspects. First, the large scale of data results in significant storage and processing challenges, requiring efficient solutions. Second, the diverse sources and varying formats of data necessitate flexible management strategies. Third, the imperative for data security and privacy protection demands the enforcement of stringent management measures. Therefore, a robust data management platform is needed to deliver comprehensive solutions for efficient storage, rapid retrieval, and secure sharing of the multi-omics and multimodal data in life sciences, to advance the progress of scientific research and clinical applications.

The Genome Sequence Archive (GSA) [9] serves as a repository for raw sequence data in the National Genomics Data Center, China National Center for Bioinformation (CNCB-NGDC) [10,11], which has effectively addressed the longstanding challenges related to the unified submission and management of genomic data in China, promoting the sharing and reuse of data within the community. In 2021, the Genome Sequence Archive family (GSA family) [12], comprising GSA, the Genome Sequence Archive for Human (GSA-Human), and the Open Archive for Miscellaneous Data (OMIX), was introduced, greatly enhancing the management and sharing capabilities for multi-omics data.

Over the past few years, the GSA family has made significant efforts to address the challenges posed by the rapid accumulation of multi-omics and multimodal data. First, the GSA family has expanded the range of data types it handles, adding biomedical imaging and clinical data, which are now archived in a new member database called the Open Biomedical Image Archive (OBIA) [13]. Through this platform, researchers can access and integrate data from various domains more comprehensively. In particular, the combination of raw sequence data with clinical and medical imaging data enables researchers to delve deeper into the molecular mechanisms of diseases, thereby providing crucial support for the development of personalized treatment plans. Second, the GSA family has comprehensively upgraded the core functionalities of its existing databases, including improvements in data quality control, search tools, data sharing mechanisms, and security measures. These improvements not only enhance the accuracy and efficiency of data processing but also ensure secure and convenient data sharing and access. In particular, optimizing the quality control process has significantly improved data precision and reliability, thereby providing standardized, high-quality datasets suitable for artificial intelligence (AI)-driven analyses. Additionally, the GSA family has mirrored and integrated raw sequence data from the National Center for Biotechnology Information (NCBI), offering unified retrieval and download services for both submitted and integrated datasets. This initiative has made cross-platform data integration smoother, facilitated the sharing and analysis of large-scale datasets, and supported research and innovation in the life sciences.

## Data model

**Figure 1:**
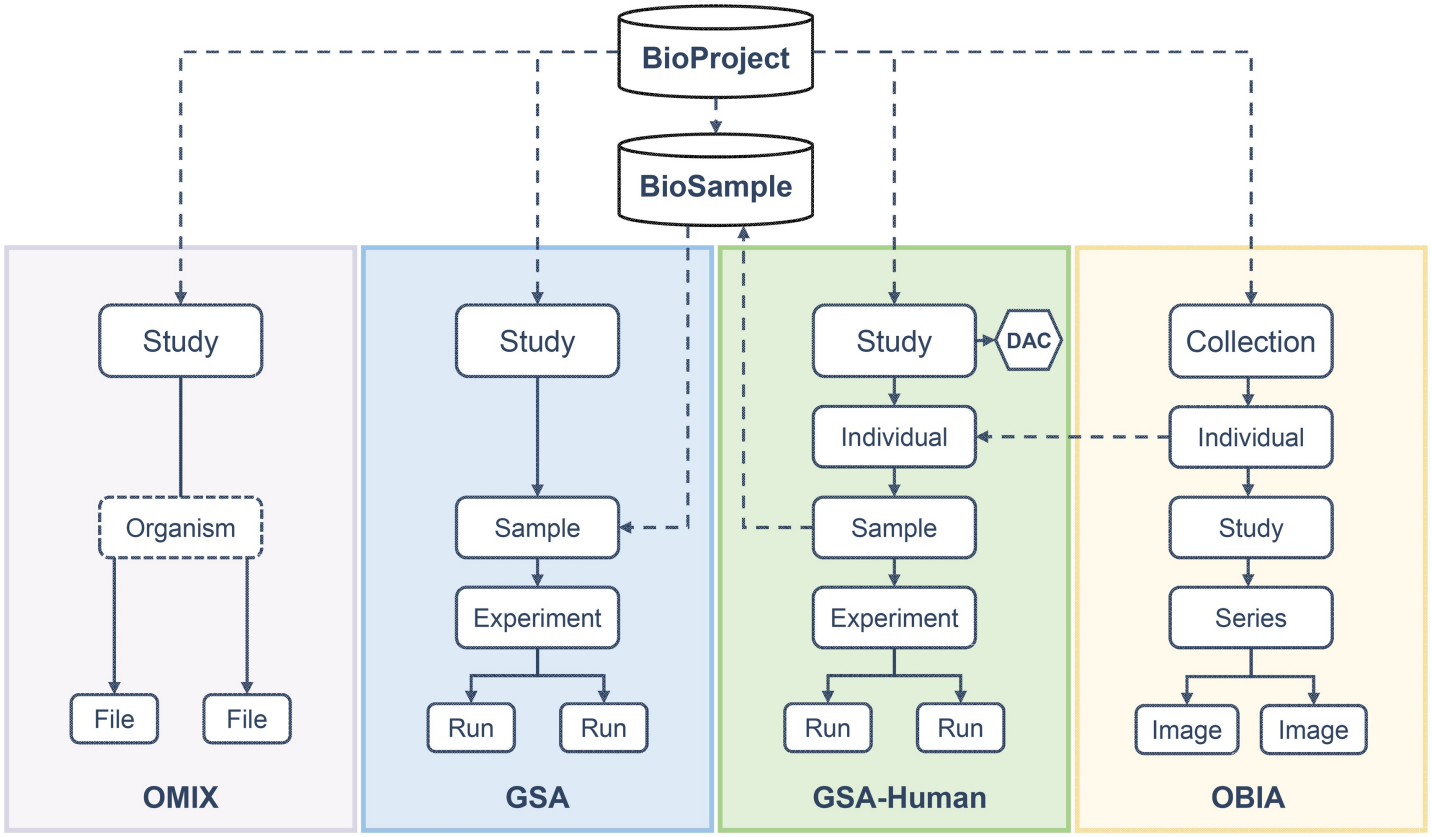
Data model of the GSA family Within the GSA family, GSA, GSA-Human, OMIX, and OBIA are closely interconnected through a BioProject accession, thereby enabling project-level integration of heterogeneous datasets. Both GSA and GSA-Human are connected to BioSample, but they don’t share sample records. GSA directly uses the BioSample database to register and manage sample information, while GSA-Human synchronizes its de-identified sample records with BioSample to support secure cross-database linkage. Both GSA-Human and OBIA use the “Individual” to organize the sequence or imaging data for an individual person, and the “Individual” in OBIA can be associated with GSA-Human by a “Individual” accession number, which links imaging data with genomic data for researchers to perform multimodal analysis. To ensure compliant access, GSA-Human allows users to form a configurable DAC by adding designated members; the DAC reviews and authorizes data access. GSA, Genome Sequence Archive; GSA-Human, Genome Sequence Archive for Human; OMIX, Open Archive for Miscellaneous Data; OBIA, Open Biomedical Imaging Archive; DAC, Data Access Committee.

As of 2025, the GSA family has expanded to four member databases: GSA, GSA-Human, OMIX, and OBIA. These databases are tightly integrated through BioProject (**Figure 1**), forming a comprehensive multi-omics data framework that ensures seamless data sharing.

In GSA, metadata is organized into four objects: Study, Sample, Experiment, and Run. Each study is identified by a BioProject (https://ngdc.cncb.ac.cn/bioproject), which serves as a key metadata repository providing structured storage for project descriptions. Sample metadata is standardized via the BioSample database (https://ngdc.cncb.ac.cn/biosample), where each sample is assigned a unique accession number to ensure data consistency and integrity, while supporting efficient sample management, convenient retrieval, and cross-database linkage.

GSA-Human adopts a structured data model comprising five core objects: Study, Individual, Sample, Experiment, and Run, and establishes an individual-centric metadata management system. It defines its own sample metadata standard based on this framework and does not rely on the BioSample database for sample management. To ensure cross-database compatibility, however, non-sensitive sample metadata is synchronized daily with BioSample, and each sample is assigned a unique BioSample accession number. This mechanism opens the possibility for other CNCB-NGDC resources, such as Genome Warehouse [14] and Genome Variation Map [15], to utilize GSA-Human metadata, while preserving GSA-Human’s distinct management approach.

OBIA organizes its data into five core objects: Collection, Individual, Study, Series, and Image, and accepts biomedical images spanning a wide range of modalities, anatomical sites, and disease types. Notably, individuals in this framework can be linked to GSA-Human via individual accessions, facilitating the integration of imaging and raw sequencing data for multi-omics research.

OMIX uses a lightweight data model with three objects: Study, Organism, and File, providing flexibility and scalability to accommodate diverse researcher submission needs. By integrating with external datasets through BioProject accessions, OMIX serves as a key complement to the GSA family. It supports specialized scientific data types that are related to, but fall outside the scope of, other member databases.

## Archival resources

### Genome Sequence Archive

GSA, developed in accordance with the data standards of the International Nucleotide Sequence Database Collaboration (INSDC) [16], is an open-access repository dedicated to archiving, retrieving, and sharing non-human raw sequence data. In 2023, GSA was selected by the Global Biodata Coalition as one of the Global Core Biodata Resources, demonstrating its pivotal role in the international landscape of biological data.

To improve user experience and streamline data access, GSA has launched a new retrieval system designed to meet the diverse needs of researchers. This advanced retrieval interface enables users to conduct sophisticated queries across multiple search fields, encompassing organism, file type, sequencing platform, sequencing strategy, and numerous other criteria. The system further empowers users by allowing them to construct intricate queries using logical operators, facilitating the precise targeting and swift retrieval of desired data. Once search results are obtained, users can apply a range of filters to further refine their outcomes. This flexibility ensures that researchers can quickly zero in on the most relevant datasets. In addition, GSA provides a convenient batch download tool that allows users to download search results in multiple formats, thereby catering to diverse requirements for information acquisition.

In addition, GSA has integrated a taxonomic classification tool built on the Sequence Taxonomic Analysis Tool (STAT) [17]. The tool performs species classification analysis on raw sequencing data and presents the results via an interactive online visualization interface. It helps researchers assess data quality and validity from a taxonomic perspective, thereby supporting the efficient selection and acquisition of high-quality datasets for their research.

### Genome Sequence Archive for Human

GSA-Human is a data repository dedicated to archiving, managing, and sharing raw sequence data from human biomedical research endeavors. GSA-Human provides two types of data access modalities: open access and controlled access. Open access allows unrestricted data downloads, while controlled access requires authorization from the Data Access Committee (DAC), as designated by the data submitter. Furthermore, GSA-Human has formulated comprehensive policies governing the sharing of human genetic resources data (https://ngdc.cncb.ac.cn/gsa-human/policy), providing users with clear guidelines on data submission procedures, management practices, and sharing protocols. These measures ensure both the security of sensitive data and its efficient utilization by the scientific community.

In recent years, the data volume in GSA-Human has experienced an exponential surge, representing a staggering sevenfold increase over 2021 levels. Notably, the most rapid expansion of 1 petabyte (PB) was achieved within a mere two days, posing significant challenges to data quality control procedures. To mitigate performance bottlenecks and improve data processing efficiency, we upgraded the quality control algorithm for FASTQ files by optimizing the data structure and reducing memory consumption. In conventional quality control workflows, duplicate READ detection is typically performed using a hash table to store all encountered READ names. However, as the file size increases, this approach results in substantial memory overhead, significantly hindering the program’s ability to process compressed files larger than 200 GB. To improve scalability and memory efficiency, our algorithm integrates a Bloom Filter (false-positive rate = 1 × 10^−9^) that utilizes bit arrays to efficiently detect duplicate READs, thereby enhancing the performance and robustness of the quality control process when handling large-scale sequencing datasets. The results indicate that the new algorithm reduces memory usage by 15 times, enabling the processing of terabyte-scale compressed files, and has been successfully integrated into GSA-Human and other relevant systems for processing raw sequence data.

In addition, GSA-Human has implemented a scoring system to evaluate the degree of openness for the published datasets. Open access datasets automatically receive the full scores, while for controlled access datasets, a comprehensive evaluation incorporates various factors related to the DAC’s handling of data access requests. These factors include the percentages of processed and approved requests, and the average processing time for the requests, which together contribute to determining the final score. This scoring methodology offers an intuitive measure of dataset sharing levels, furnishing data requesters with clear and understandable insights. It is our hope that such a scoring system will foster a culture of data sharing and encourage the reuse of scientific data. The scoring details for the public datasets are available online (https://ngdc.cncb.ac.cn/gsa-human/browse).

### Open Archive for Miscellaneous Data

OMIX, built in adherence to the Findable, Accessible, Interoperable, and Reusable (FAIR) principles [18], stands as a versatile data repository, dedicated to collecting, publishing, and sharing scientific data for biological researchers. Currently, OMIX supports ten primary data types, including 32 subcategories that span functional genomics, proteomics, metabolomics, and more (Table S1), highlighting its flexibility and scalability for diverse and evolving research needs.

OMIX offers two distinct data access options: open access and controlled access. Both are available for human genetic resource data, while data from other species are limited to open access. To manage and authorize controlled data access, OMIX employs a mechanism similar to that of GSA-Human, but with a simplified ‘request and approval’ process. This allows data requesters to easily submit applications and enables data providers to approve requests with a single click, thereby reducing processing time and improving the efficiency of data acquisition.

As a significant member of the GSA family, OMIX broadens the scope of data intake and enhances data diversity. By linking to other member databases through BioProject IDs, it creates multi-omics datasets centered on raw sequencing data from GSA or GSA-Human, offering robust support for advancing scientific research. For instance, BioProject PRJCA006118 hosts 25 paired multi-omics datasets from the Chinese Multi-omics Advances in Sepsis (CMAISE) project, a multi-omics research project focusing on sepsis. The CMAISE project collects clinical data and biological samples from sepsis patients and applies multi-omics technologies such as genomics, proteomics, and metabolomics to comprehensively investigate the molecular mechanisms and clinical characteristics of sepsis. Its publication of eight research papers has not only deepened the understanding of sepsis pathophysiology but also suggested potential directions for its clinical treatment.

### Open Biomedical Imaging Archive

OBIA, the new member of the GSA family, serves as an innovative data repository meticulously crafted to store and share multimodal medical imaging alongside its clinical data. It boasts a dual-pronged approach to data accessibility, encompassing both open and controlled access mechanisms, catering to varying user needs and security requirements. This feature highlights OBIA’s pivotal role in fostering collaborative research by bridging the gap between diverse data modalities and facilitating the synthesis of insights for advanced medical discoveries.

The primary challenge in constructing a medical image database is ensuring robust privacy protection. Images may contain protected health information (PHI) and must be properly processed before being archived to minimize the risk of patient privacy breaches. OBIA addresses this by providing a unified de-identification and quality control mechanism based on the Digital Imaging and Communications in Medicine (DICOM) standard. The key elements and rules we follow include: (1) removing sensitive pixel data, (2) cleaning descriptive metadata, (3) retaining modified dates for longitudinal temporal information, (4) preserving patient characteristics, (5) retaining device identifiers, and (6) keeping safe private tags. OBIA utilizes the Radiological Society of North America (RSNA) MIRC clinical trial processor (CTP) (https://mircwiki.rsna.org/index.php?title=MIRC_CTP) to remove or blank certain standard tags containing or potentially containing PHI. For private tags, we employ PyDicom (https://pypi.org/project/pydicom) to retain attributes that are purely numeric. Furthermore, images with PHI in their pixel data are rigorously filtered to safeguard patient privacy. The complete de-identification pipeline is detailed in our earlier publication [13].

In addition to providing web interfaces for data query and browsing, OBIA offers innovative image retrieval capabilities [19]. The retrieval model is based solely on image features and uses the Hamming distance to return visually similar images. Users can upload images to search the database for the top 30 most similar images, significantly enhancing the user experience.

## Data submission

The GSA family is equipped with the Single Sign-On (SSO) service, a user access control system that enables seamless authentication across multiple databases with a single, unified ID and password. To submit data to the GSA family, users are required to register an account through the SSO system and log in to the specific database where they intend to submit their data.

Each database in the GSA family provides a user-friendly web-based submission service that defines a series of metadata templates tailored to the characteristics of the accepted data, facilitating the collection of the required information. For example, GSA-Human offers specialized templates for human-related research, covering diverse areas such as disease, cohort, cell line, clinical pathogen, and human-associated metagenome studies. The templates cover essential attributes required for the data objects, such as sample details, experimental conditions, and sequencing strategies, while also allowing users to incorporate custom attributes, greatly enhancing adaptability to personalized research needs.

In addition, each database in the GSA family has implemented a rigorous quality control system and a manual curation process to ensure the accuracy and reliability of submitted data. Particular attention is paid during the manual review stage to identifying any content that may involve personal privacy or sensitive information. If such issues are identified, submitters are promptly notified to make the necessary revisions. This mechanism not only ensures data compliance but also establishes a strong foundation for the sharing and reuse of high-quality scientific data.

## Data mirroring

The INSDC maintains the largest collection of raw sequence data, which is not readily accessible to many researchers in China, mainly due to the large volume and limited network bandwidth. The three member databases of INSDC, Sequence Read Archive (SRA), DNA Data Bank of Japan (DDBJ), and European Nucleotide Archive (ENA), conduct data exchange and sharing on a daily routine. As of December 2024, SRA contains more than 28 PB of published data, compared to 7.5 PB in GSA. To enable seamless sharing and reuse of global data, GSA has developed a system to mirror raw sequence data from INSDC, sourced via NCBI. Metadata, including BioProjects, BioSamples, and SRAs, is downloaded, parsed and stored locally in sync, and the corresponding sequence files are mirrored accordingly. All metadata and newly released sequence files since April 2022 are mirrored and synchronized daily, ensuring consistency with INSDC. Furthermore, all mirrored data are integrated into GSA and equipped with retrieval, browsing, and download functions, providing users with convenient access. The integrated GSA, which consolidates data from multiple sources, offers a broader range of information than other similar databases, providing users with a more comprehensive and informative resource.

## Data statistics

**Figure 2:**
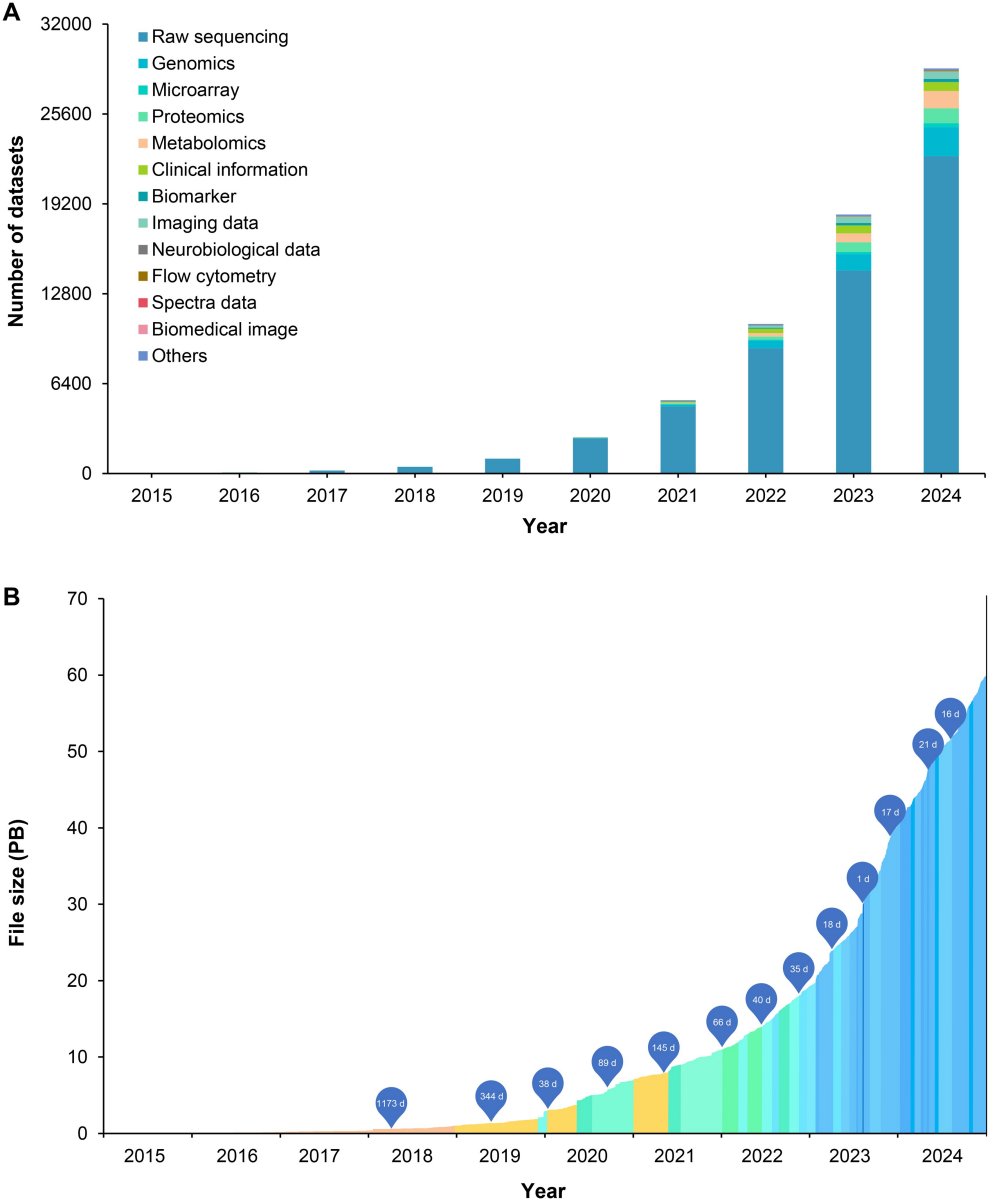
Data statistics of the GSA family **A**. The cumulative number of datasets submitted to the GSA family from 2015 to 2024, categorized into 13 major data types. **B**. The growth in the volume of raw sequence data submissions over time, with the duration required to accumulate each additional petabyte (PB) indicated. All the statistics are based on data from GSA and GSA-Human as of December 2024. The numbers within the bubbles indicate the number of days elapsed for the data volume to increase by 1 PB. PB, petabyte; d, day.

As of December 2024, the GSA family has archived 28,838 datasets (**Figure 2**A), totaling more than 59.98 PB of data. From 2015 to 2024, there has been a remarkable upsurge in both the volume and diversity of data. The data volume has grown exponentially, and the diversity of data types has significantly increased (Figure 2B; **Table 1**). Data accessions from the GSA family have been cited in 4351 research articles across 729 scientific journals (https://ngdc.cncb.ac.cn/gsa/statistics?active=journals). For detailed statistics on data submissions from each database, please refer to Table 1.

**Table 1:** Data submissions within the GSA family

Data item	GSA	GSA-Human	OMIX	OBIA	Total
**Archival statistics**					
Dataset count	16,001	6602	6229	6	28,838
BioProject count	13,583	5888	4035	3	23,509
Individual count	/	582,817	/	1094	583,911
Sample count	889,841	847,958	/	/	1,737,799
Experiment count	891,329	952,690	/	/	1,844,019
Run count	965,023	1,248,968	/	/	2,213,991
File count	1,744,888	1,943,316	32,094	2,012,007	5,732,305
Data volume (TB)	19,722.24	41,605.12	90.97	0.39	61,418.72
**Statistics by access method**					
Open-access dataset count	16,001	1559	2925	1	20,486
Controlled-access dataset count	0	5043	3304	5	8352
**Publication statistics**					
Support publication count	2497	1037	974	/	4351

*Note*: All the statistics were derived from the GSA family as of December 2024. “/” means not applicable. Due to the overlaps that occur when individual articles cite accession numbers from multiple databases, the total number of unique articles is smaller than the sum of article counts across the databases. GSA, Genome Sequence Archive; GSA-Human, Genome Sequence Archive for Human; OMIX, Open Archive for Miscellaneous Data; OBIA, Open Biomedical Imaging Archive; TB, terabyte.

Regarding data sharing, the GSA family offers two main access options: open and controlled access. Through open access, the database provides researchers with unrestricted access to datasets. As of December 2024, the GSA family has published 14,558 open-access datasets, totaling 7.45 PB of data shared via FTP and HTTP, with 166,549,212 total downloads. For controlled access, the GSA family has published 4756 datasets, received 9383 access requests from 4829 users, and approved 3,242 of them, resulting in nearly 10 PB of data downloads. Detailed statistics on data sharing for each database are provided in **Table 2**. As of December 2024, GSA has mirrored 783,566 projects, 41,867,651 samples, 32,507,936 experiments, and 34,456,823 runs, along with more than 10.8 PB of sequence files from NCBI.

**Table 2:** Data sharing within the GSA family

Data item		GSA	GSA-Human	OMIX	OBIA	Total
All published datasets	**Archival statistics**					
	Dataset count	11,174	4008	4126	6	19,314
	**Data sharing metrics**					
	Share count	162,698,110	3,798,674	55,665	5	166,552,454
	Download volume (TB)	5757.50	12,045.08	48.97	0.48	17,852
Open-access datasets	**Archival statistics**					
	Dataset count	11,174	1168	2215	1	14,558
	**Data sharing metrics**					
	Download count	162,698,110	3,795,803	55,299	/	166,549,212
	Download volume (TB)	5757.50	1852.66	16.00	/	7,626.16
Controlled-access datasets	**Archival statistics**					
	Dataset count	/	2840	1911	5	4756
	**Data sharing metrics**					
	Requester count	/	4082	728	19	4829
	Request count	/	8470	885	28	9383
	Approval count	/	2871	366	5	3242
	Download volume (TB)	/	10,192.42	32.97	0.48	10,225.87

*Note*: All the statistics were derived from the GSA family as of December 2024.

## Future direction

The GSA family has continually improved its archival databases for raw sequence, multi-omics, and multimodal data, streamlining data submission, management, and reuse. Unlike conventional archives, this four-member archive family supports a broader spectrum of data types across diverse species and research fields. Its innovative management approach, focused on raw omics data, enables efficient multi-omics integration and delivers a comprehensive suite of services to users.

Future efforts will be focused on the ongoing optimization of data models and management processes to meet evolving user needs and rapidly growing data volumes. An advanced cloud-based data storage architecture, along with a suite of innovative tools, will be developed to enhance the efficiency of storing and mining of the omics big data. To address the challenges posed by the exponential growth of data, a more automated data management system will be developed to streamline the entire workflow, covering data submission, reception, quality control, data archiving, publication, and updating, thereby improving overall processing efficiency.

For the secure and sustainable management of human genetic resource data, a comprehensive strategy will be employed. This includes the implementing or enhancing of data classification frameworks, management mechanisms, reliable backup and recovery systems, and advanced anonymization and encryption technologies. In parallel, efforts will be made to explore a secure cloud-based data analysis model, which is expected to reduce privacy risks associated with the direct download of raw data. By utilizing a controlled storage environment for sensitive files, this model significantly reduces the potential for data exposure. Furthermore, the development and implementation of individual identification techniques based on genomic sequences will also be actively pursued.

Additionally, active efforts are underway to promote global collaborations focused on advancing data standards, technologies, and methodologies. Concurrently, work is being conducted to achieve full mirroring and synchronization of data from INSDC, along with the integration of additional global omics database resources. These initiatives are designed to support the widespread sharing and utilization of global biodiversity and health-related big data.

## Supplementary Material

qzaf072_Supplementary_Data

## Data Availability

GSA is open accessible at https://ngdc.cncb.ac.cn/gsa [12]. GSA-Human is freely accessible at https://ngdc.cncb.ac.cn/gsa-human [12]. OMIX is freely accessible at https://ngdc.cncb.ac.cn/omix [12]. OBIA is freely accessible at https://ngdc.cncb.ac.cn/obia [13].

## CRediT author statement

**Sisi Zhang:** Investigation, Methodology, Data curation, Writing – original draft, Writing – review & editing. **Xu Chen:** Software, Writing – original draft. **Enhui Jin:** Software, Investigation, Data curation, Writing – original draft. **Anke Wang:** Software, Writing – original draft. **Tingting Chen:** Investigation, Methodology, Data curation, Writing – original draft. **Xiaolong Zhang:** Software, Writing – original draft. **Junwei Zhu:** Software. **Lili Dong:** Data curation. **Yanling Sun:** Data curation. **Caixia Yu:** Data curation. **Yubo Zhou:** Software. **Zhuojing Fan:** Software. **Huanxin Chen:** Resources. **Shuang Zhai:** Resources. **Yubin Sun:** Resources. **Qiancheng Chen:** Resources. **Jingfa Xiao:** Conceptualization. **Shuhui Song:** Conceptualization. **Zhang Zhang:** Conceptualization. **Yiming Bao:** Conceptualization. **Yanqing Wang:** Conceptualization, Investigation, Methodology, Software, Writing – review & editing. **Wenming Zhao:** Conceptualization, Methodology, Writing – review & editing, Supervision. All authors have read and approved the final manuscript.

## ORCID

0000-0002-3852-4796 (Sisi Zhang)

0000-0001-6102-1751 (Xu Chen)

0009-0000-9916-9508 (Enhui Jin)

0000-0002-2565-2334 (Anke Wang)

0000-0003-1296-3093 (Tingting Chen)

0000-0002-9968-9818 (Xiaolong Zhang)

0000-0003-4689-3513 (Junwei Zhu)

0000-0003-0953-6306 (Lili Dong)

0000-0002-3175-3625 (Yanling Sun)

0000-0002-3882-9979 (Caixia Yu)

0009-0008-1697-3110 (Yubo Zhou)

0009-0003-3575-6967 (Zhuojing Fan)

0000-0003-1293-4495 (Huanxin Chen)

0000-0002-2084-7132 (Shuang Zhai)

0000-0003-3810-7156 (Yubin Sun)

0009-0005-5675-3858 (Qiancheng Chen)

0000-0002-2835-4340 (Jingfa Xiao)

0000-0003-2409-8770 (Shuhui Song)

0000-0001-6603-5060 (Zhang Zhang)

0000-0002-9922-9723 (Yiming Bao)

0000-0002-7985-7941 (Yanqing Wang)

0000-0002-4396-8287 (Wenming Zhao)
