# Supplementary material for: The GSA Family in 2025: A Broadened Sharing Platform for Multi-omics and Multimodal Data
Source: Genomics Proteomics Bioinformatics. 2025 Aug 26;23(4):qzaf072. doi: 10.1093/gpbjnl/qzaf072 (PMC12451262; doi:10.1093/gpbjnl/qzaf072)
Supplement: qzaf072_Supplementary_Data [file qzaf072_supplementary_data.zip › Supplementary material captions.docx]

# Supplementary materials

**Table S1 Data type of OMIX database**
