# Supplementary material for: The GSA Family in 2025: A Broadened Sharing Platform for Multi-omics and Multimodal Data
Source: Genomics Proteomics Bioinformatics. 2025 Aug 26;23(4):qzaf072. doi: 10.1093/gpbjnl/qzaf072 (PMC12451262; doi:10.1093/gpbjnl/qzaf072)
Supplement: qzaf072_Supplementary_Data [file qzaf072_supplementary_data.zip › Table S1.docx]

## Table S1 Data type of OMIX database

| **Data type** | **Sub type** | **Datasets** | **File size (TB)** |
| --- | --- | --- | --- |
| Genomics | Methylation Profiling by NGS | 2073 | 20.35 |
|  | Expression Profiling by NGS |  |  |
|  | Non-coding RNA Profiling by NGS |  |  |
|  | Genome Binding/Occupancy Profiling by NGS |  |  |
|  | Chromatin Accessibility Profiling by NGS |  |  |
|  | Other Type of Genomic Data |  |  |
| Microarray | Methylation profiling by Array | 282 | 0.26 |
|  | Expression Profiling by Array |  |  |
|  | Non-coding RNA Profiling by Array |  |  |
|  | Genome binding/occupancy profiling by Array |  |  |
|  | Other type of Microarray Data |  |  |
| Proteomics | Protein 3D Structure Data | 1048 | 39.19 |
|  | Proteomic Data by Mass Spectrometry |  |  |
|  | Other Type of Proteomic Data |  |  |
| Metabolomics | Metabolome Data by Mass Spectrometry | 1231 | 19.81 |
|  | Lipidome Data by Mass Spectrometry |  |  |
|  | Other Type of Metabolome Data |  |  |
| Clinical Information | Demographic Data | 638 | 0.92 |
|  | Clinical Research Data |  |  |
|  | Other Type of Clinical Information |  |  |
| Biomarker | Genetic Biomarkers | 535 | 0.90 |
|  | Karyotype Biomarkers |  |  |
|  | Protein Biomarkers |  |  |
|  | Chemical Biomarkers |  |  |
|  | Condition-specific Biomarkers |  |  |
| Imaging | Magnetic Resonance Imaging | 228 | 4.65 |
|  | Behavioral Video Data |  |  |
|  | Other Type of Image Data |  |  |
| Neurobiological Data | / | 30 | 0.75 |
| Flow cytometry | / | 51 | 0.13 |
| Spectra Data | Raman Spectra Data | 10 | 0.02 |
|  | Other type of Spectra Data |  |  |
| Others | / | 103 | 3.99 |

## *Note*: All statistics regarding data sharing were derived from the OMIX as of December 2024. “/” means not applicable. TB, terabyte. Before 2022, OMIX allowed multiple data types in one dataset. To improve usability, it was refined to 10 major categories and 32 subcategories. Data once labelled “Other” (*e.g.*, imaging, molecular spectra, protein sequences, CT scans, biomarkers) are now integrated into these new categories, and “Other” is no longer used.
